# The impact of depression in Alzheimer’s disease hospitalized patients: a study protocol for a nationwide retrospective study

**DOI:** 10.1192/j.eurpsy.2022.670

**Published:** 2022-09-01

**Authors:** B. Brás, A.R. Ferreira, M. Gonçalves-Pinho, A. Freitas, L. Fernandes

**Affiliations:** 1 University of Porto, Faculty Of Medicine, Porto, Portugal; 2 Faculty of Medicine, University of Porto, Department Of Clinical Neurosciences And Mental Health, Porto, Portugal; 3 Faculty of Medicine, University of Porto, Cintesis – Center For Health Technology And Services Research, Porto, Portugal; 4 Faculty of Medicine, University of Porto, Department Of Community Medicine, Information And Health Decision Sciences (medcids), Porto, Portugal; 5 Centro Hospitalar do Tâmega e Sousa, Department Of Psychiatry And Mental Health, Penafiel, Portugal; 6 Psychiatry Service, Centro Hospitalar Universitário De São João, Porto, Portugal

**Keywords:** Alzheimer’s disease, Depression, Administrative Database, Hospitalization outcomes

## Abstract

**Introduction:**

Alzheimer’s disease (AD) is the leading cause of dementia worldwide. About 40-50% of AD patients are also affected by depression, with mounting evidence suggesting its association with worse disease prognosis and negative outcomes, such as lower quality of life, higher mortality and more hospitalizations. However, few studies have specifically measured the association of depression with AD hospitalization outcomes.

**Objectives:**

To characterize depression among all hospitalizations with a registered diagnosis of AD and to explore its association with hospitalization outcomes, including in-hospital mortality, length of stay and discharge destination.

**Methods:**

A retrospective observational study will be conducted following the RECORD statement. A Portuguese nationwide hospitalization database from all mainland public hospitals will be used. Episodes of inpatients ≥65 years old with a primary or secondary diagnosis of AD (ICD-9-CM code 331.0), discharged between 2008-2015, will be selected. Codes 296.2X, 296.3X, 300.4 and 311 will be used to identify episodes with a concomitant registry of depression at any diagnostic position. Descriptive, univariate and multivariate approaches will be used.

**Results:**

A total of 61 361 episodes complying with the fixed criteria will be assigned to one of two groups (with vs without depression). Groups will be compared regarding sociodemographic characteristics, comorbidity profile, type of admission (planned vs urgent) and hospitalization outcomes. Results regarding the association of depression and outcomes will be presented as crude and adjusted odds ratios (OR).

**Conclusions:**

With this nationwide analysis, we expect to contribute to the clarification of depression impact on AD hospitalizations, so that best-practice care may be provided to these patients.

**Disclosure:**

No significant relationships.

